# Supplementary figures and images for: Formation of 1-octen-3-ol from Aspergillus flavus conidia is accelerated after disruption of cells independently of Ppo oxygenases, and is not a main cause of inhibition of germination
Source: PeerJ. 2014 May 20;2:e395. doi: 10.7717/peerj.395 (PMC4034645; doi:10.7717/peerj.395)

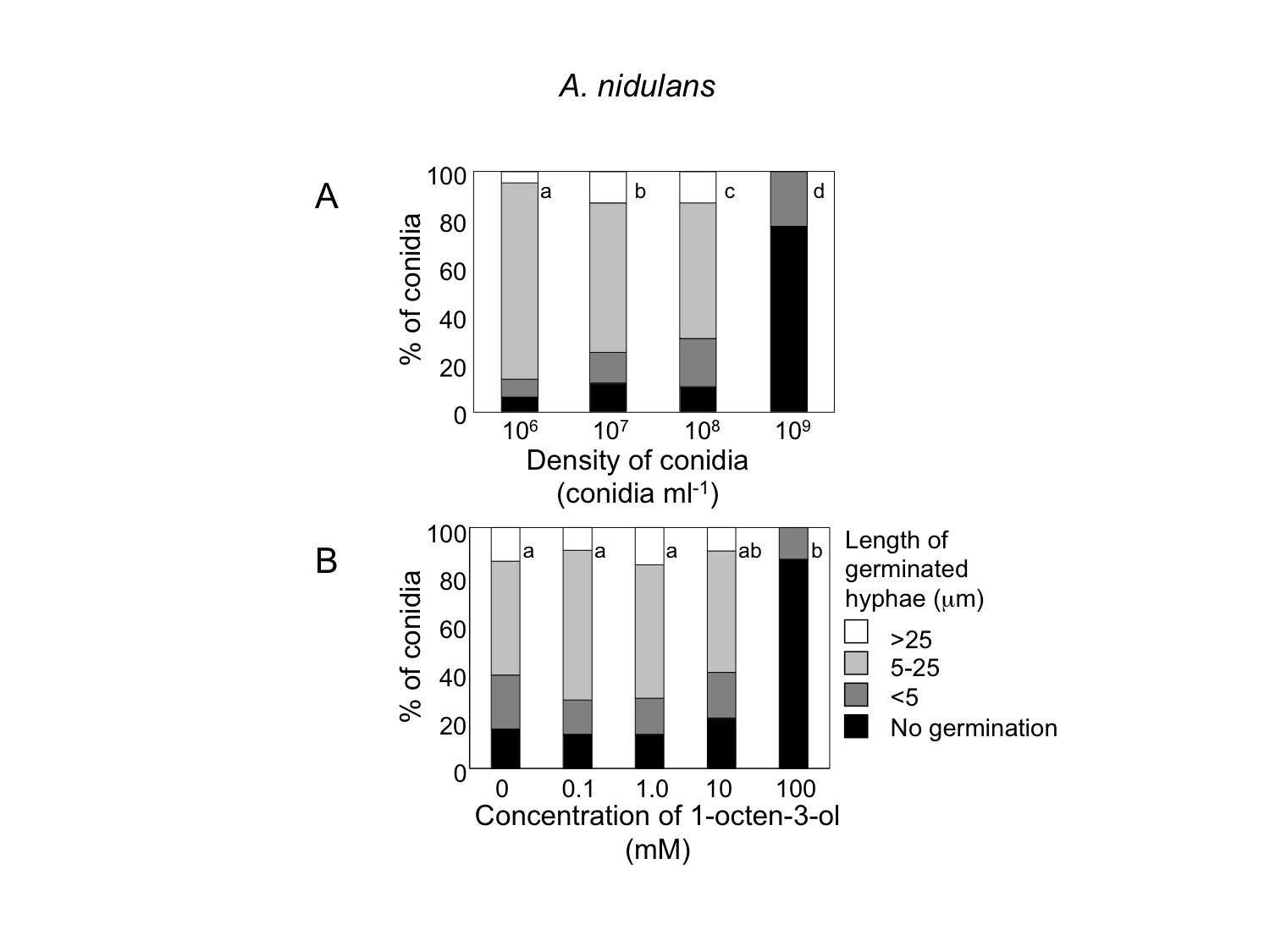

Supplement: Figure S1 — (A) Conidia of A. nidulans were prepared from 1-week old GMM plates, then, resuspended in GMM at 1.0 × 10^6^ to 1.0 × 10^9^ spores mL−1. The suspensions were incubated at 29 °C for 9 h, then, the germination rate and the length of hyphae of germinated conidia were examined under microscope. (B) To the conidia set at 1.0 × 10^6^ spores mL−1, 1-octen-3-ol was added. Germination of conidia was examined as above. Different letters indicate statistically significant differences (P < 0.05, Kruskal-Wallis, n = 200). [file peerj-02-395-s001.png]
